# Evolution Meets Disease: Penetrance and *Functional Epistasis* of Mitochondrial tRNA Mutations

**DOI:** 10.1371/journal.pgen.1001379

**Published:** 2011-04-21

**Authors:** Raquel Moreno-Loshuertos, Gustavo Ferrín, Rebeca Acín-Pérez, M. Esther Gallardo, Carlo Viscomi, Acisclo Pérez-Martos, Massimo Zeviani, Patricio Fernández-Silva, José Antonio Enríquez

**Affiliations:** 1 Departamento de Bioquímica y Biología Molecular y Celular, Universidad de Zaragoza, Zaragoza, Spain; 2 Departamento de Bioquímica, Instituto de Investigaciones Biomédicas “Alberto Sols,” Facultad de Medicina, CSIC–Universidad Autónoma de Madrid, CIBERER, ISCIII, Madrid, Spain; 3 Division of Molecular Neurogenetics, Istituto Neurologico “Carlo Besta,” Milano, Italy; 4 Regenerative Cardiology Department, Centro Nacional de Investigaciones Cardiovasculares Carlos III, Madrid, Spain; Max Planck Institute for Biology of Aging, Germany

## Abstract

About half of the mitochondrial DNA (mtDNA) mutations causing diseases in humans occur in tRNA genes. Particularly intriguing are those pathogenic tRNA mutations than can reach homoplasmy and yet show very different penetrance among patients. These mutations are scarce and, in addition to their obvious interest for understanding human pathology, they can be excellent experimental examples to model evolution and fixation of mitochondrial tRNA mutations. To date, the only source of this type of mutations is human patients. We report here the generation and characterization of the first mitochondrial tRNA pathological mutation in mouse cells, an m.3739G>A transition in the mitochondrial *mt-Ti* gene. This mutation recapitulates the molecular hallmarks of a disease-causing mutation described in humans, an m.4290T>C transition affecting also the human *mt-Ti* gene. We could determine that the pathogenic molecular mechanism, induced by both the mouse and the human mutations, is a high frequency of abnormal folding of the tRNA^Ile^ that cannot be charged with isoleucine. We demonstrate that the cells harboring the mouse or human mutant tRNA have exacerbated mitochondrial biogenesis triggered by an increase in mitochondrial ROS production as a compensatory response. We propose that both the nature of the pathogenic mechanism combined with the existence of a compensatory mechanism can explain the penetrance pattern of this mutation. This particular behavior can allow a scenario for the evolution of mitochondrial tRNAs in which the fixation of two alleles that are individually deleterious can proceed in two steps and not require the simultaneous mutation of both.

## Author Summary

Mitochondrial DNA (mtDNA) encodes for 13 proteins that are subunits of the Oxidative Phosphorylation system. These proteins are synthesized within the mitochondria by its own set of ribosomes. In mammals the rRNAs and all the tRNAs required for the mitoribosomes to work are also encoded by the mtDNA. Surprisingly, half of mtDNA mutations causing diseases occur in tRNA genes while they represent only 10% of the total sequence. Moreover, these pathological tRNA mutations can occur in homoplasmic form (all the copies of the mtDNA are mutant). It is puzzling that, with these homoplasmic mutations, some individuals suffer a severe disease while others are healthy. Recently we have described that common variants of mtDNA in mouse induce differences in the performance of the Oxidative Phosphorylation capacity but that the cell is capable to sense these differences and adapt the biogenesis of the OXPHOS system to compensate for them. We also showed that this mechanism was triggered by differences in the basal level of ROS. It was considered that this adaptation mechanism was of no relevance in pathological mutations. The implication of the ROS-mediated mitochondrial biogenesis in modulating the consequences of mild pathogenic mutations and its significance for disease penetrance and mitochondrial tRNA evolution are underscored.

## Introduction

Mammalian mitochondrial DNA is a double-stranded circular molecule that codes for 13 of the 87 proteins that constitute the OXPHOS system, as well as two rRNAs and the 22 tRNAs required for mitochondrial protein synthesis. Mutations in mitochondrial DNA are known to be responsible for a wide variety of diseases in humans whose common characteristic is the impairment of the OXPHOS system. Almost half of the ≈250 mutations described so far are located within tRNA genes [1]. All tRNA genes are affected in at least one position, being *mt-Tl1* the most represented with 23 different reported mutations followed by *mt-Tk* and *mt-Ti* with 15 and 14 mutated positions respectively.

Cells carrying pathological mutations in mt-tRNAs usually exhibit impaired respiration and reduced growth rates in medium with galactose instead of glucose. This is due to the fact that mutations in tRNA genes may affect the synthesis of critical subunits of Complexes I, III and IV and two subunits of complex V. Different mutations produce a variety of defects [2] including impaired aminoacylation [3]–[5], reduced tRNA half-life [6], impairment of pre-tRNA processing [7]–[10], decrease in the steady-state levels of tRNA [11] and others, promoting, therefore, protein synthesis deficiency. Very often, however, when mitochondrial protein synthesis activity is directly estimated by metabolic labeling in cultured cell models, no decrease in overall protein synthesis rate can be detected [12]–[23]. This is particularly problematic when studying homoplasmic pathological tRNA mutations with an unexplained partial penetrance of the disease [23].

Mutations in mt-tRNAs tend to promote different disease patterns. Thus, while different mutations in *mt-Tk* cause MERRF or MERRF-like syndromes [2], [24]–[26], mutations in *mt-Ti*, usually have cardiomyopathy as the main or one of the cardinal features [2], [27], [28]. On the other hand, deafness is one of the prominent symptoms associated with tRNA mutations [2]. However, the picture is more complex since it is now clear that different mutations can raise similar disease phenotypes while in other cases the same mutation, such as m.3243G>A, can promote very different diseases.

In mice, there is no description of mitochondrial tRNA pathological mutations. However, it has been reported the existence of four different alleles in a highly polymorphic loci at the *mt-Tr* [29]. Interestingly, although none of these alleles is pathological by itself, they modulate the expression of the age-associated hearing loss due to a Cadherin 23 mutation [30]. Moreover, these alleles are associated with variable ROS production by mitochondria and with a different performance of the mitochondrial respiratory chain. We described also a ROS-mediated mitochondrial biogenesis compensatory response associated with non-pathological variants of mouse mtDNA that serve for the fine-tuning of the OXPHOS capacity of the cells [31]. This mechanism was also triggered by pathological mutations affecting protein coding genes, but without significant benefit since enhancing biogenesis of a deleterious mtDNA would be detrimental rather than compensatory [31].

We describe here the first pathological mutation in a mouse mitochondrial tRNA, an m.3739G>A transition in the mitochondrial *mt-Ti* gene. The mutation is located in the anticodon loop of this tRNA, two bases downstream from the anticodon, and generates a new potential Watson and Crick pair between the first and last base of the loop. Interestingly, we previously described an analogous mutation in humans, a homoplasmic T to C transition two bases upstream the mt-tRNA^Ile^ anticodon triplet, responsible for a progressive necrotizing encephalopathy with variable penetrance [16]. We found that both, the human and the mouse mutations, promote a similar structural deficiency in the mt-tRNA^Ile^ that causes a reduction in the effective amount of functional isoleucyl-tRNA^Ile^. As a consequence, mitochondrial protein synthesis and the activity of complexes I, III and IV are impaired, causing a mild but significant OXPHOS deficiency. We describe also that cells harboring the mutant mtDNA show a higher ROS production that leads to a compensatory response to this respiration deficiency by enhancing mitochondrial biogenesis. This response is able to partially compensate the deficiency. Therefore we demonstrate the positive implication of the ROS-mediated mitochondrial biogenesis also in the expression of mitochondrial tRNA pathological mutations found in human patients. These observations highlight the different nature of the mutations affecting protein-coding genes vs. tRNA genes with consequences to our understanding of pathology and evolution of mitochondrial tRNAs. Thus, this mechanism may generate an epistatic-like effect (“functional epistasis”) by which a partial suppression of deleterious mutations in mitochondrial tRNAs is exerted. This increased mitochondrial biogenesis may allow the survival and reproduction of some individuals despite of harboring a deleterious allele, facilitating the appearance of a true compensatory mutation, the bona-fide epistatic mutation.

## Results

### Isolation of a mitochondrial tRNA defective mouse cell line

**Figure 1:**
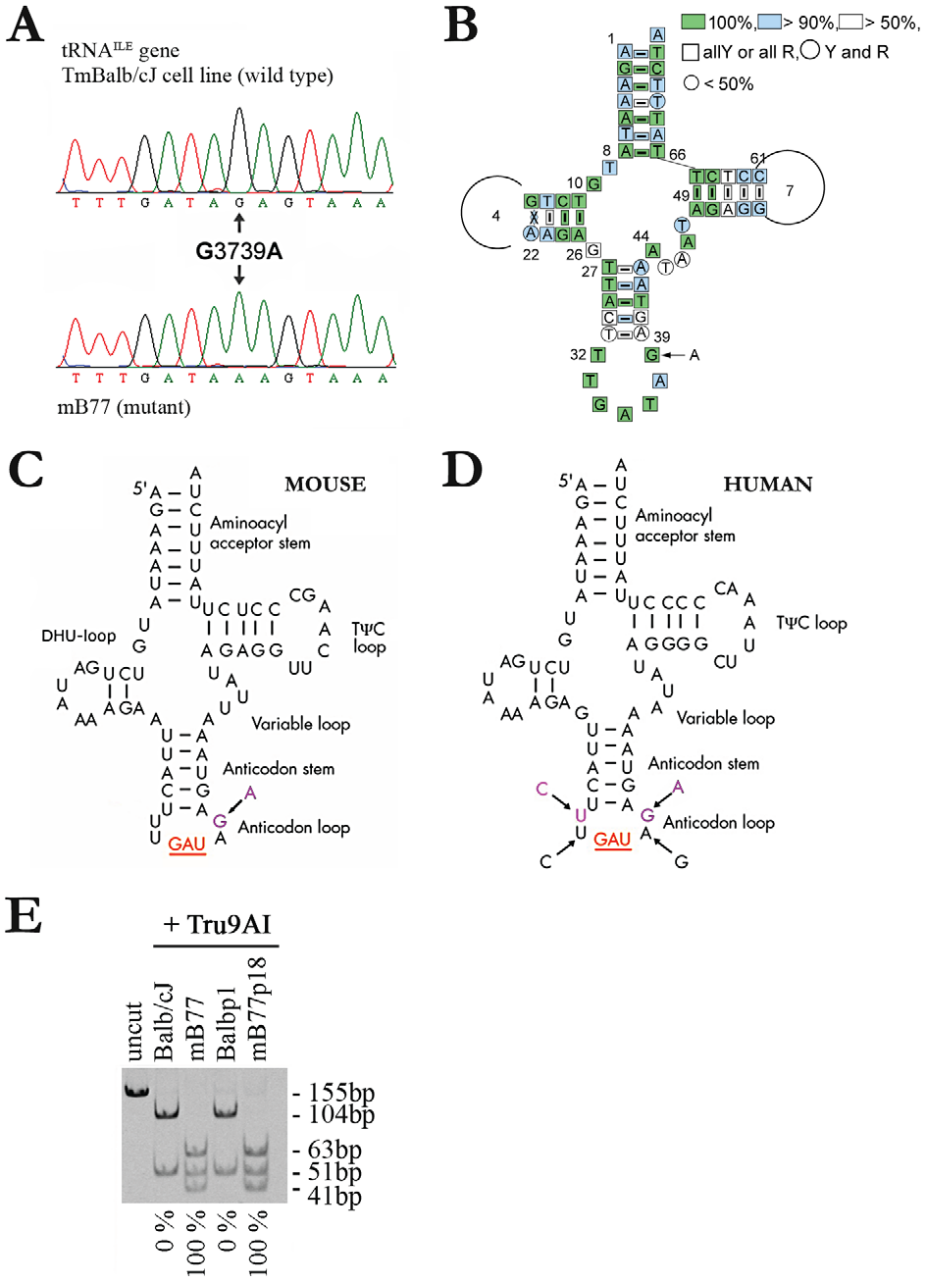
Characterization of the *mt-Ti* mutation. A) Chromatogram showing the m.3739G>A mutation within the *mt-Ti* gene in mB77 cells. B) Conservation of mt-tRNA^Ile^ primary sequence in 150 studied mammals (http://mamit-trna.u-strasbg.fr, 2007). Percentage indicates the degree of conservation, Y (pyrimidines) R (purines). C, D) Proposed secondary structure of the tRNA^Ile^ in mouse (C) and human cells (D). The mutant nucleotides replace the wild-type ones in the anticodon loop (arrows). Wild-type and mutant nucleotides are represented in magenta and the GAU anticodon sequence is in red. Other mutations previously reported within the anticodon loop, are shown (D). E) RFLP analysis of the m.3739G>A mutation in both mB77 and the transmitochondrial clone mB77p18. The presence of the mutation creates an extra recognition site for Tru9I.

In our laboratory, we systematically induce and isolate mtDNA mutations by random mutagenesis using different mitochondrial backgrounds [32], [33]. In this case, mutagenesis was performed in the cell line TmBalb/cJ, obtained by transfer of mitochondria from Balb/cJ mouse platelets to mtDNA-depleted cells ρ°L929^neo^ [34] and hence carrying the mtDNA of Balb/cJ. In this way, we isolated a potential OXPHOS defective clone, mB77. In order to securely assess the mtDNA responsibility of the phenotype observed, we performed mitochondrial transfer from mB77 to a different cell line lacking mtDNA, ρ°L929^puro^ (the transmitochondrial cell line thus generated was called mB77p). Then, we fully sequenced the mtDNA of these cell lines and we found a unique mutation, consisting in an m.3739G>A transition affecting the *mt-Ti* gene (Figure 1A). This nucleotide, 100% conserved in 150 species of mammals (Figure 1B), is located at the tRNA anticodon loop, two bases downstream from the anticodon (Figure 1B and 1C). An identical mutation (m.4296G>A) has been found in humans associated with a degenerative encephalopathy (Rossmanith, W, personal communication) and in oncocytic tumors [35]. Interestingly, a homoplasmic T to C mutation two bases upstream the mt-tRNA^Ile^ anticodon triplet (m.4290T>C), has been described in humans and it could also promote a new potential Watson and Crick pair between the first and last base of the anticodon loop (Figure 1D). The latter mutation was also associated with a degenerative encephalopathy [16]. Homoplasmic or near homoplasmic mutant cell lines were obtained by long-term culturing of mB77 heteroplasmic cells, or by subcloning of mB77p (mB77p18). This was confirmed by RFLP analysis (Figure 1E). Finally, TmBalb/cJ mitochondria were also transferred to ρ°L929^puro^ cells (Balbp1) in order to generate a proper control for mB77p18 cells.

### m.3739G>A mutation promotes defective respiration rates and impaired growth in galactose

**Figure 2:**
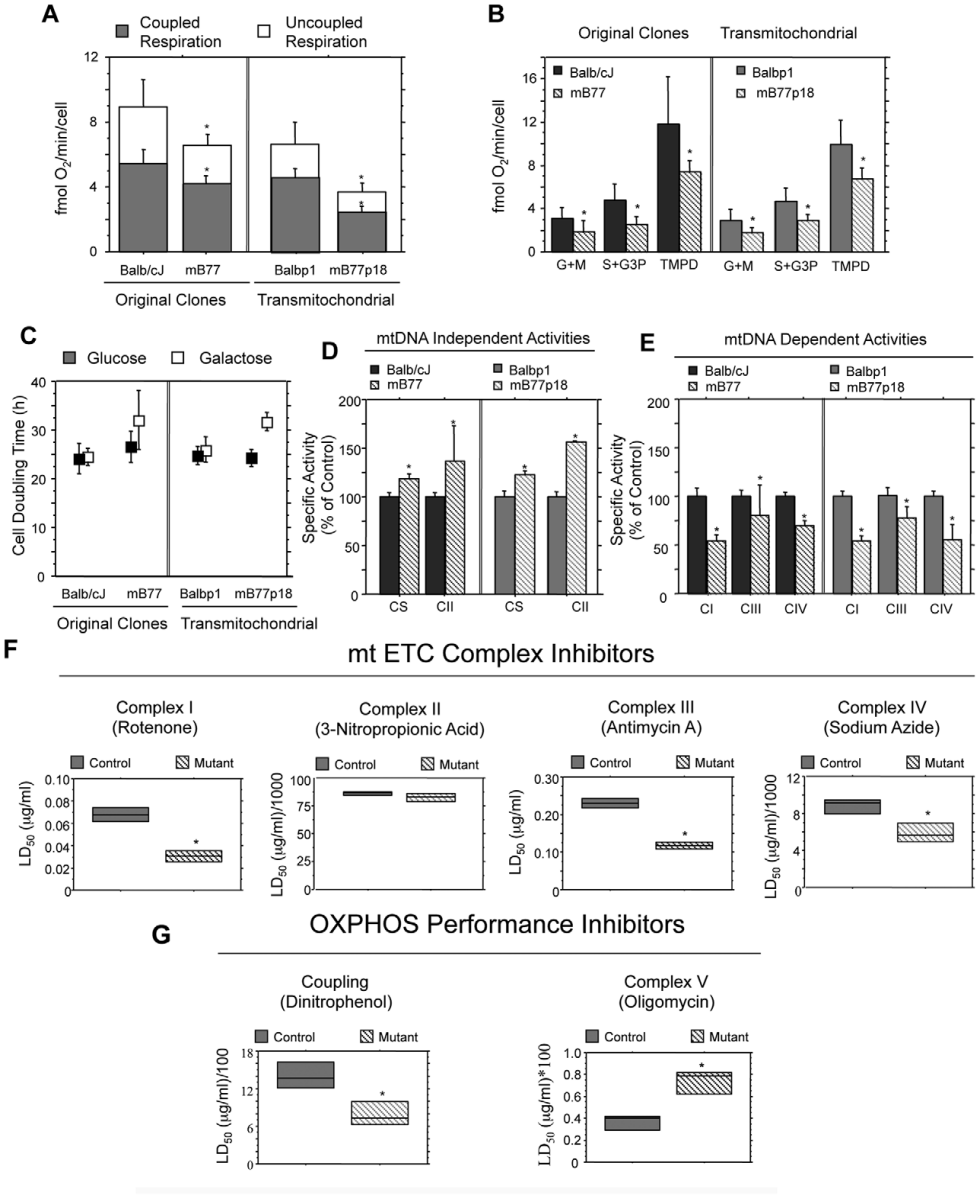
Functional analysis of OXPHOS performance. A) Oxygen consumption rate in intact cells (n = 11, 9, 13 and 12 for TmBalb/cJ, mB77, Balbp1 and mB77p18 respectively). B) Oxygen consumption of permeabilized cells in the presence of electron donors for complex I (Glutamate + Malate), complex III (Succinate + G3P) and complex IV (TMPD) (n = 8 in all cases except for mB77p18 where n = 9). C) Growth ratio (doubling time in hours, DT) for each cell line, in a medium containing galactose and in a medium containing glucose (see Materials and Methods for details; n = 7, 5, 5 and 3 for TmBalb/cJ, mB77, Balbp1 and mB77p18 respectively). D) Spectroscopic measurement of mtDNA independent activities: citrate synthase (CS) and Complex II, in mutant and wild type cells lines (n = 3 in all cases). E) Spectroscopic measurement of isolated mitochondrial complexes I, III and IV activities in mutant and control cell lines (n≥3 in all cases). F) Estimation of the LD_50_ for the indicated inhibitors of the different respiratory complexes in control (TmBalb/cJ) and mutant (mB77) cells (n = 3 in both control and mutant for all inhibitors but antimycin A where n = 2; p = 0.0426 for rotenone, p = 0.3022 for 3-Nitropropionic Acid, p = 0.0181 for Antimycin A and p = 0.0460 for sodium azide). G) Evaluation of the LD_50_ for the indicated inhibitors of OXPHOS performance in control (TmBalb/cJ) and mutant (mB77) cells (DNP: n = 4 for control cells and n = 3 for mutants, p = 0.0278 and oligomycin: n = 3 and p = 0.0201). All values are given as mean ± SD of the mean. Asterisks indicate significant differences respect to each control, tested by ANOVA post-hoc Fisher PLSD (p<0.05).

As shown in Figure 2A, cell respiration was significantly decreased in the mutant cell lines (an average reduction of 24% in mB77 and 46% in mB77p18, relative to the respective control cell line). Compatible with a tRNA mutation, Figure 2B illustrates that the reduction in respiration is maintained at any entry point of the electrons, suggesting an affectation of the whole respiratory chain (see below).

Cells with impaired OXPHOS capacity show difficulties to grow in medium where glucose is substituted by galactose as carbon source, and this depends very much on the extent of the OXPHOS impairment [36], [37]. Figure 2C shows how cells carrying the Balb/cJ mtDNA display a similar doubling-time (DT) in glucose and in galactose (ratio DT Gal/Glu for TmBalb/cJ, 1.018 and for Balbp1, 1.044). On the contrary, cells harboring mutant mtDNAs present a significant delayed growth in galactose with respect to glucose (ratio DT Gal/Glu for mB77, 1.201 and for mB77p18, 1.304).

### The m.3739G>A mutation reduces the activity of all respiratory complexes with mtDNA-encoded subunits

A hallmark of pathological mt-tRNA mutations is that usually all respiratory complexes with mtDNA encoded subunits are affected [2]. Here, while mitochondrial enzymes with no mtDNA encoded subunits (citrate synthase and complex II) showed a small but significant increase in activity in mutant cells (Figure 2D), all the respiratory complexes harboring subunits encoded by mtDNA showed a significant activity reduction (Figure 2E).

To independently assess the OXPHOS performance at the different enzymatic steps, we estimated the sensitivity of cell survival to drugs that affect the function of specific respiratory complexes (Figure 2F), the ATPase and the coupling between respiration and ATP synthesis (Figure 2G). Thus, the lethal dose 50 (LD_50_) was significantly smaller for mutant versus wild type tRNA^Ile^ cells for complex I, III and IV inhibitors while the only respiratory complex without mtDNA encoded subunits, complex II, was equally affected by its inhibitor in control and mutant cells (Figure 2F). In addition, mutant cells were also more sensitive to uncouplers (Figure 2G). On the contrary, mutant cells were more resistant to ATP synthase inhibition by oligomycin (Figure 2G), suggesting that they have an excess of ATP synthesis capacity with respect to proton gradient generation capacity.

### Mitochondrial biogenesis is enhanced in mt-tRNA^Ile^ mutant cells

**Figure 3:**
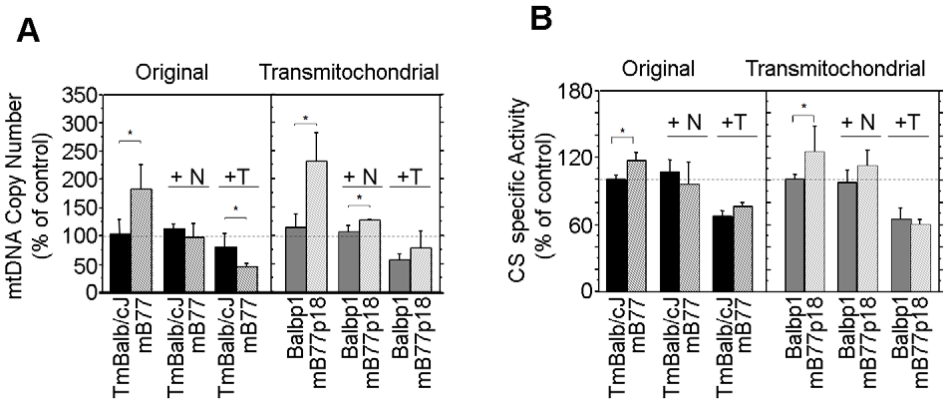
Analysis of the biogenetic response induced by the mutation and effect of ROS scavengers. A) mtDNA copy number variation between wild type and mutant cells without drugs (left) or in the presence of NAC (center) or Tiron (right) (n = 25, 22, 14 and 12 for TmBalb/cJ, mB77, Balbp1 and mB77p18 respectively and p<0.0001 between each mutant and its control in the absence of scavengers; n = 3 in all cases but mB77 (n = 4) and p = 0.0376 between Balbp1 and mB77p18 in the presence of NAC and n = 7, 3, 5 and 4 for TmBalb/cJ, mB77, Balbp1 and mB77p18 respectively after treatment with Tiron; p = 0.0406 between TmBalb/cJ and mB77). B) Spectroscopic measurement of specific citrate synthase activities without drugs (left) and after treatment with NAC (center) or Tiron (right) (n = 8 in all cell lines but mB77 (n = 6); p<0.0001 between TmBalb/cJ and mB77 and p = 0.0081 between Balbp1 and mB77p18 in the absence of scavengers, n = 4 in all cases in the presence of NAC and n = 3 for all cell lines after treatment with Tiron). N = NAC and T = Tiron.

Both, the increase in citrate synthase and Complex II activities, suggested that mitochondrial mass and, therefore, mitochondrial biogenesis could be increased in mutant cells. This was confirmed by determining the amount of mtDNA per cell as a robust index of mitochondrial biogenesis. Thus, mutant cells almost double the amount of mtDNA with respect to control cells and have significantly higher citrate synthase (CS) specific activities (Figure 3 and Figure S1A). Recently, we have demonstrated that elevated production of H_2_O_2_ by mitochondria triggers the signaling cascade that adapts mitochondrial biogenesis to cell demands [31]. In agreement with this, we also found that cells carrying the m.3739G>A mutation produce more H_2_O_2_ (Figure S1B) and that ROS scavengers such as N-Acetyl-Cysteine (NAC) or Tiron added to the culture medium, abolish the signal that triggers mitochondrial biogenesis and equalizes the level of mtDNA and CS activity between mutant and wild type cells (Figure 3 and Figure S1C). As a consequence of this, cell respiration decreased significantly more in mutant than in control cells after the NAC treatment indicating an effective compensation of the defect by the biogenesis activation (Figure S1D).

### Mitochondrial protein synthesis is impaired in cells carrying m.3739G>A mutation

**Figure 4:**
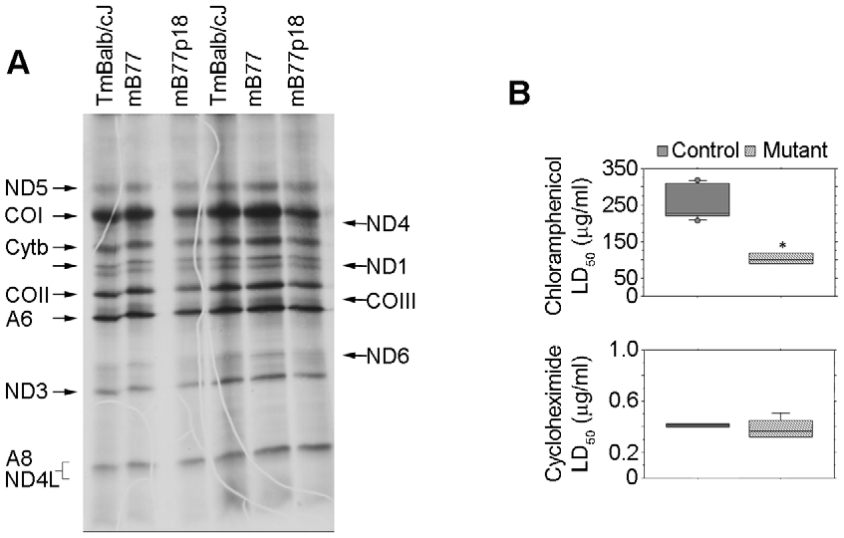
Analysis of mitochondrial protein synthesis and protein synthesis sensitivity to inhibitors. A) Fluorogram, after electrophoresis through an SDS-polyacrylamide gradient gel, of the mitochondrial translation products of the mutant and wild-type cells, labeled with [^35^S]-methionine for 1 hr in the presence of emetine (ND1 to 6: NADH dehydrogenase subunit 1 to 6; Cytb: Cytochrome b; COI, II and III: Cytochrome C Oxidase subunits I to III; A6 and A8: ATP synthase subunits 6 and 8). B) Differential influence of CAP and cycloheximide in wild-type (TmBalb/cJ) versus mutant (mB77) cells viability (n = 7 for control and n = 3 for mutant cells in the case of CAP (p = 0.0009) and n = 4 and 5 for control and mutant respectively in the case of cycloheximide (p = 0.5948**)**. All values are given as mean ± SD of the mean. Asterisks indicate significant differences respect to each control, tested by ANOVA post-hoc Fisher PLSD (p<0.05).

The fact that the m.3739G>A mutation affects a tRNA gene strongly suggests that the observed OXPHOS phenotype could be explained by an impairment in mitochondrial protein synthesis. To investigate this, *in vivo* protein synthesis experiments were performed. Analysis of mitochondrial DNA translation products did not reveal significant quantitative differences or abnormal migration of any polypeptide in the mutant cells (Figure 4A). This puzzling observation is, however, very commonly reported in other pathological tRNA mutations and suggests that the drop of mitochondrial protein synthesis should be very severe to be revealed by this methodology. Nevertheless, one has to be aware that the experiment shown in Figure 4A reflects the rate of mitochondrial protein synthesis on a “per cell” basis. Then, if the amount of mtDNA per cell is taken into consideration, a less efficient use of the mitochondrial genome would be revealed in mutant cells. In order to get a more sensitive approach, we reasoned that if mitochondrial protein synthesis was in fact impaired in the mutant cells they would become more sensitive to specific inhibitors of mitochondrial protein synthesis such as chloramphenicol (CAP). When we analyzed this possibility, we found that, the LD_50_ for CAP was significantly lower for cells carrying m.3739G>A mutation when compared with wild type cells (Figure 4B, upper panel). This does not reflect a general weakness of the cell since the LD_50_ for cycloheximide, a specific inhibitor of the cytoplasmic ribosomes, remains similar in both cell types (Figure 4B, lower panel).

### Mitochondrial respiratory complexes assembly is reduced in cells with m.3739G>A mutation

**Figure 5:**
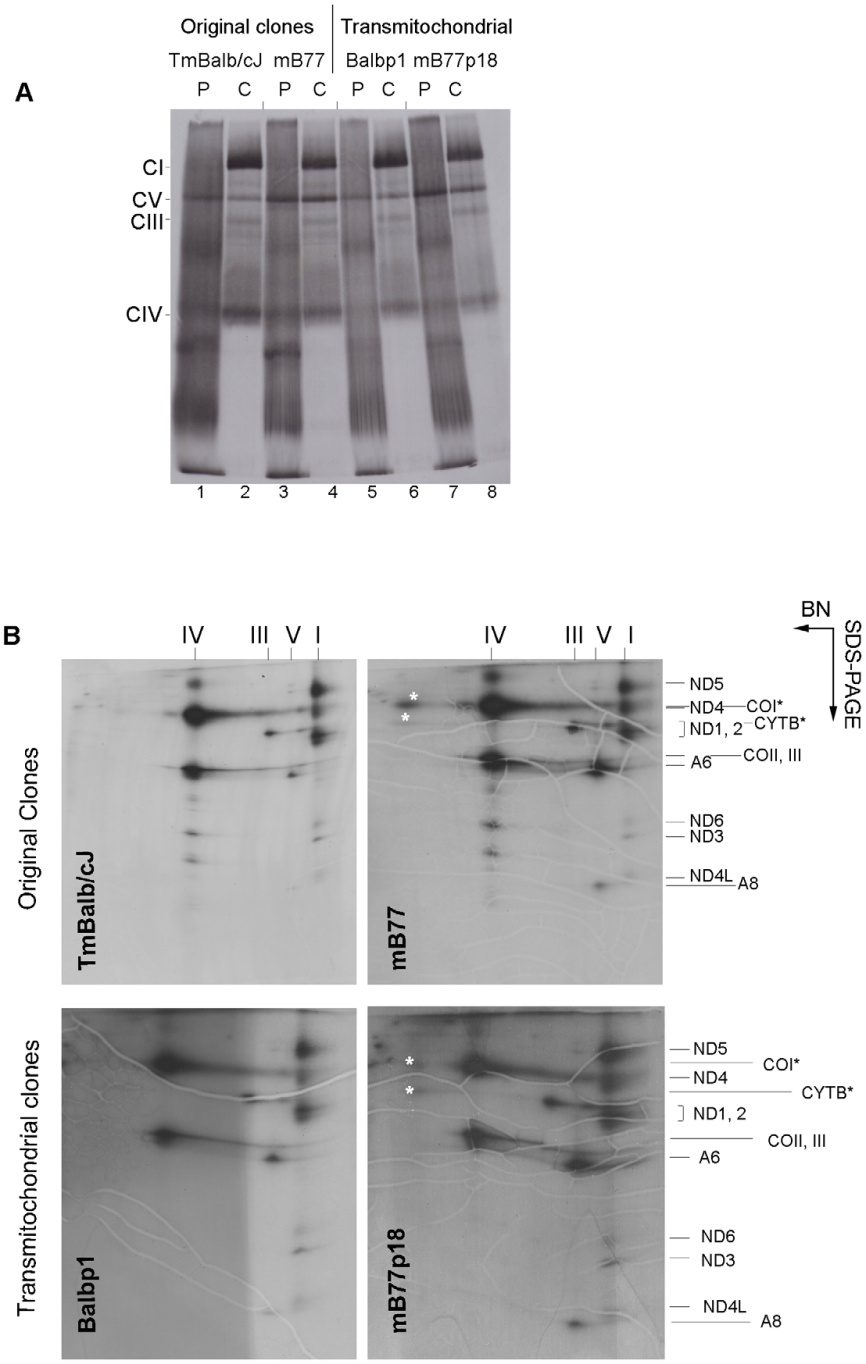
Metabolic labeling of the assembled OXPHOS complexes. A) Fluorogram, after BNGE, of the mitochondrial translation products of mutant and control cells, pulse-labeled with [^35^S]-methionine for 2 hr in the presence of cycloheximide (P) and chased (C) for 48 hours; CI-CV, complexes I to V. B) Fluorograms of two-dimensional electrophoresis (BNGE followed by SDS-PAGE), of the mitochondrial translation products obtained in b (48h chase). I-V, indicate the position of complexes I to V. Asterisks show the presence of low molecular weight subcomplexes containing CYTB and COI in mutant cell lines.

In an attempt to further investigate the consequences of the protein synthesis impairment induced by the m.3739G>A mutation, we performed *in vivo* metabolic labeling of the mitochondrial-encoded proteins followed by a chase period of 48 hours. Blue Native gel electrophoresis (BNGE) analysis (Figure 5A) revealed that the amount of the assembled complexes is affected in mutant cells where complexes I, IV, and likely complex III, seemed to be reduced. On the contrary, and in agreement with the lower sensitivity to oligomycin, complex V seems to be increased in mutant cells. Thus, complex I/V ratio was decreased in both mutants to a similar level, being 21% in mB77 and 38% in mB77p18, relative to each control, while the other two complexes were diminished in a very different proportion (mB77: III/V = 23%; IV/V = 19%; mB77p18: III/V = 84.5%; IV/V = 81.7%).

Very interestingly, when BNGE was followed by SDS-polyacrylamide gel electrophoresis of the labeled products we could observe the accumulation of subcomplexes affecting mainly complexes III and IV, both in the m.3739G>A original and transmitochondrial mutants. These did not appear in the control samples (Figure 5B).

**Figure 6:**
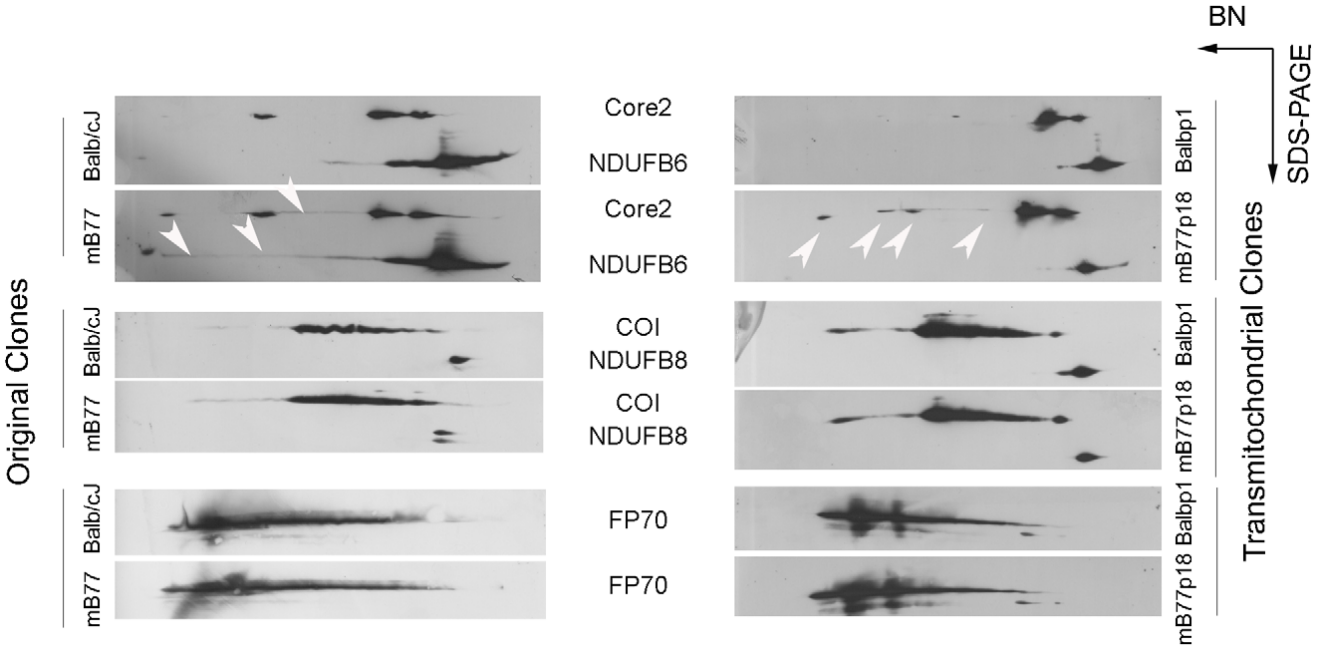
Steady-state level of respiratory complexes. Western blot of the different assembled complexes after two-dimensional electrophoresis (BNGE followed by SDS-PAGE) probed with monoclonal antibodies specific for complexes I (anti NDUFB6 and NDUFB8), III (anti Core2), IV (anti COI) and II (anti SDHA (FP70)). The presence of subcomplexes containing complex III and complex I subunits (arrowheads) is also observed in the steady state in mutant cells.

To confirm the relevance of this observation, BNGE followed by western blot was performed (Figure 6). Thus, we could detect in mutant cells the presence of subcomplexes of complexes I and III that did not appear in controls (Figure 6). Therefore, the deficiency in mitochondrial protein synthesis induced by the m.3739G>A mutation causes a disturbance in the assembly of the respiratory complexes or a reduction in their stability.

### The mt-tRNA^Ile^ amount is slightly decreased in mutant cells

**Figure 7:**
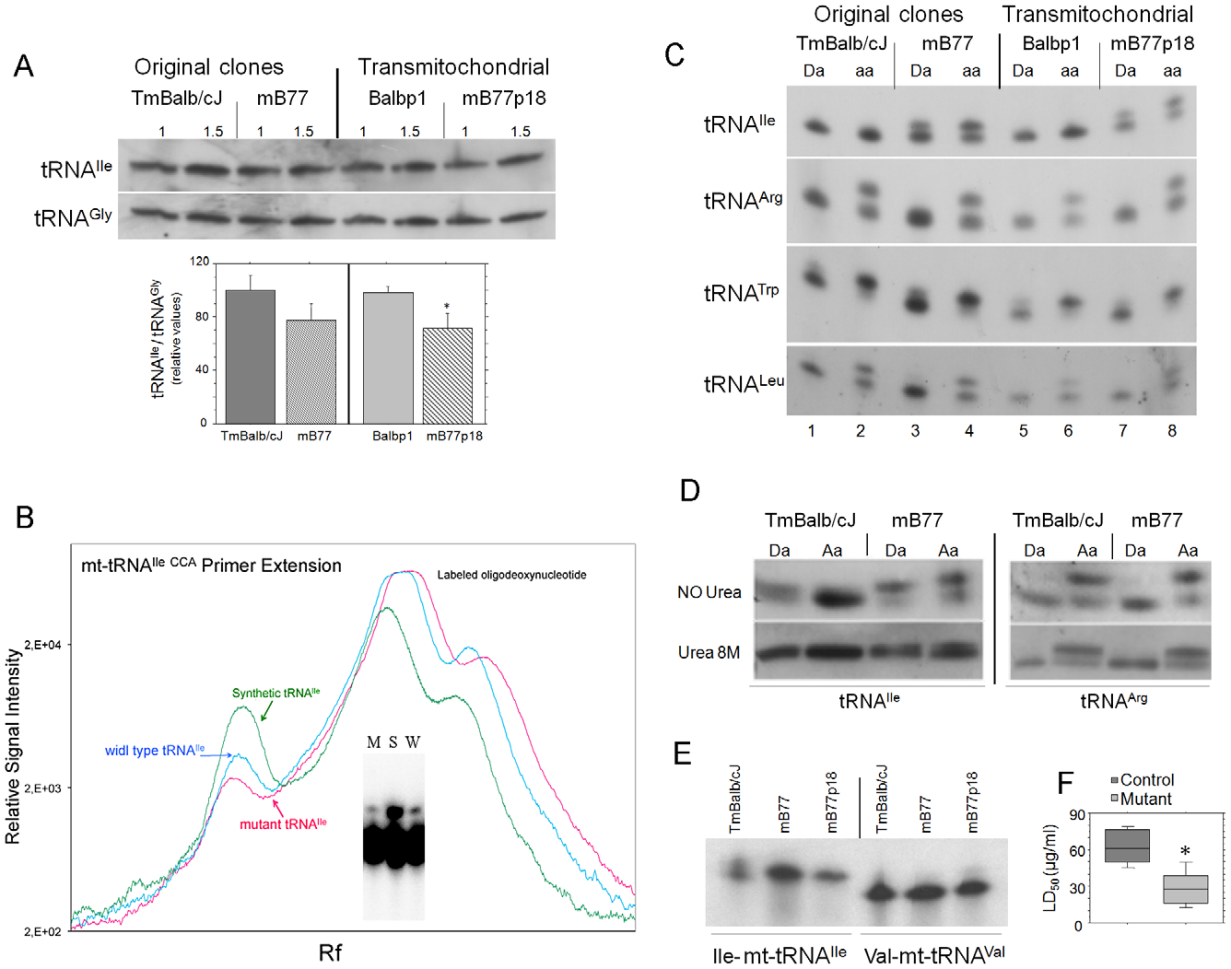
mt-tRNA analysis in *mt-Ti* mouse mutants. A) Relative tRNA^Ile^ levels in mutant and control cell lines. The radioactive signal (upper panel) obtained after hybridization of total mitochondrial RNA with the specific tRNA^Ile^ probe was normalized by the signal of the tRNA^Gly^ probe obtained from the same blot. The lower panel represents the quantification of the ratio in each cell line. B) Analysis of mt-tRNA^Ile^ precursor processing. The CCA addition to mutant and control tRNA-Ile was analyzed by allele-specific termination of primer extension. For control purposes we used synthetic mt-tRNA^Ile^ (S). The electrophoretic profiles were analyzed with the 1-D Analysis Software Quantity One. C) Analysis of the aminoacylation capacity of mutant mt-tRNA^Ile^. The identification of the lower band as the uncharged tRNA was made by running in parallel a sample of deacylated tRNA and the quality of the samples was tested by sequential hybridization with different probes specific for mt-tRNA^Arg^, mt-tRNA^Trp^ and mt-tRNA^Leu1^. D) Analysis of the electrophoretic mobility of the indicated tRNAs as in C) with no urea or with 8M urea. E) Fluorogram after electrophoresis under acidic conditions of total mt-RNA samples obtained from *in organello* aminoacylation experiments. The assays were performed in isolated mitochondria from control and mutant cell lines using either L-[4,5-^3^H]-Isoleucine or L-[3,4(n)-^3^H]-Valine. F) LD_50_ of pentamidine in control (TmBalb/cJ) vs. mutant (mB77) cells (n = 3 in both cases and p = 0.0064, ANOVA post-hoc Fisher PLSD test).

To investigate whether the mt-tRNA^Ile^ amount was diminished in our mutant cell lines, high-resolution northern blot analysis was performed. As shown in Figure 7A, the steady-state level of the mt-tRNA^Ile^ in mutant cells was almost normal, with a value of 80% of the amount in controls when normalized by tRNA^Gly^ signal. Such a small reduction would likely be of no functional significance.

### m.3739G>A mutation and mt-tRNA^Ile^ precursor processing

It has been reported that some pathogenic mutations in mt-tRNA^Ile^ affect steps in tRNA maturation including 3′-end processing and CCA addition [8], [10]. To analyze the possible effect of m.3739G>A mutation on mt-tRNA^Ile^ precursor processing, several cDNA clones derived from circularized mt-tRNA^Ile^ from wild type and mutant cell lines were sequenced [11]. Thus, 14 out of 17 sequences from control cells and 11 out of 18 from mutant cells showed the expected 3′CCA and 5′ ends. Some of the remaining sequences are likely due to artifacts where the oligodeoxynucleotide used for cDNA synthesis was ligated to the 5′-end of the tRNA. The gene encoding the mt-tRNA^Ile^ overlaps two nucleotides with the 3′ end of the *mt-Nd1* gene and three nucleotides with the 5′ end of the gene encoding for the tRNA^Gln^. We believe that RNAs derived from the processing of tRNA^Gln^ and ND1 mRNA explain the finding of this proportion of circularized products with the lack of 3′ and 5′ portions of the tRNA^Ile^. In summary, since the major proportion of molecules showed a proper maturation of the 3′ and 5′ and CCA addition, we conclude that no major defect in the processing of the mt-tRNA^Ile^ can be attributed to the mutation (Figure S2). We also confirmed the proper 3′ CCA addition in mutant mt-tRNA^Ile^ by performing allele specific termination of primer extension (Figure 7B).

### Cells harboring m.3739G>A mutation show an abnormal folding of the mt-tRNA^Ile^

In order to investigate the aminoacylation status of the mutant mt-tRNA^Ile^, mitochondrial nucleic acids were purified under acid conditions, electrophoresed through an acid (pH = 5) 10% polyacrilamide/4M urea gel and electroblotted onto a zeta-probe membrane. Then, the blots were sequentially hybridized with specific probes for different mitochondrial tRNAs. Under these conditions the acylated and deacylated forms of most of the tRNAs may migrate differently due to the induction of a conformational change in the tRNA upon aminoacylation, while an increase in urea concentration decreases the tRNA folding and minimizes the migration differences [38]. In that way we detected for tRNA^Arg^, tRNA^Trp^ and tRNA^Leu^, two bands, the slower moving corresponding to aminoacylated tRNA species (Figure 7C). The identification of the faster moving band as the uncharged tRNA was made by running in parallel a sample of deacylated tRNA [38]. Unfortunately, in the case of mt-tRNA^Ile^, the two forms, acylated and deacylated, do not separate enough to allow estimation of the aminoacylation level (Figure 7C). Interestingly, a second slower migrating band appeared only in the mutant mt-tRNA^Ile^. This second band was present even after the deacylation treatment suggesting, therefore, a second structural conformation of the uncharged tRNA. To confirm that the second band reveals, in fact, a different conformation of the tRNA, we analyzed them again either in fully native conditions (no urea) or in higher (8 M) urea concentration (Figure 7D). We confirmed that the separation between the two bands of mt-tRNA^Ile^ was almost abolished when the tRNA was closer to full denaturation (Figure 7D).

To understand whether the slower-migrating extra band in the mt-tRNA^Ile^ of mutant cells could be charged with its cognate amino acid, *in organello* aminoacylation experiments using L-^3^H-aminoacids were performed [38], [39]. Thus, we incubated isolated mitochondria in the presence of either L-[4,5-^3^H]-Isoleucine or L-[3,4(n)-^3^H]-Valine as a control. As shown in Figure 7E, when *in organello*-aminoacylated tRNAs were isolated and electrophoresed under acidic conditions, only one band was observed. These results confirm that the slower moving extra band present in mutant cell lines was not isoleucyl-tRNA^Ile^, and therefore, that it corresponds to a non-chargeable form of the tRNA^Ile^. As a consequence, the ratio of isoleucyl-tRNA^Ile^ /tRNA^Ile^ seemed to be abnormally low in the mutant cells.

In addition, since we propose the missfolding of the tRNA^Ile^ as the primary cause of the protein synthesis defect observed in mutant cells, we also tested the influence of a drug (pentamidine), that specifically targets mitochondrial tRNAs preventing their proper folding [40], on the survival of the cells. As shown in Figure 7F, cells carrying m.3739G>A mutation are significantly more sensitive to pentamidine than wild type cells.

### The human m.4290T>C and the murine m.3739G>A mutations in mtDNA cause similar molecular effects

To determine whether the observed changes in the tRNA^Ile^ secondary structure were also present in human cell lines carrying mutations in tRNA^Ile^ anticodon loop, we took advantage of human transmitochondrial cell lines harboring the m.4290T>C pathological mutation [16]. This mutation is located two bases upstream the tRNA anticodon triplet and creates a new potential Watson and Crick pair between the first and last base of the loop (Figure 1D). As a control, we used a pool of transmitochondrial cell lines obtained by transferring mitochondria from platelets of different healthy individuals belonging to different mtDNA haplogroups.

**Figure 8:**
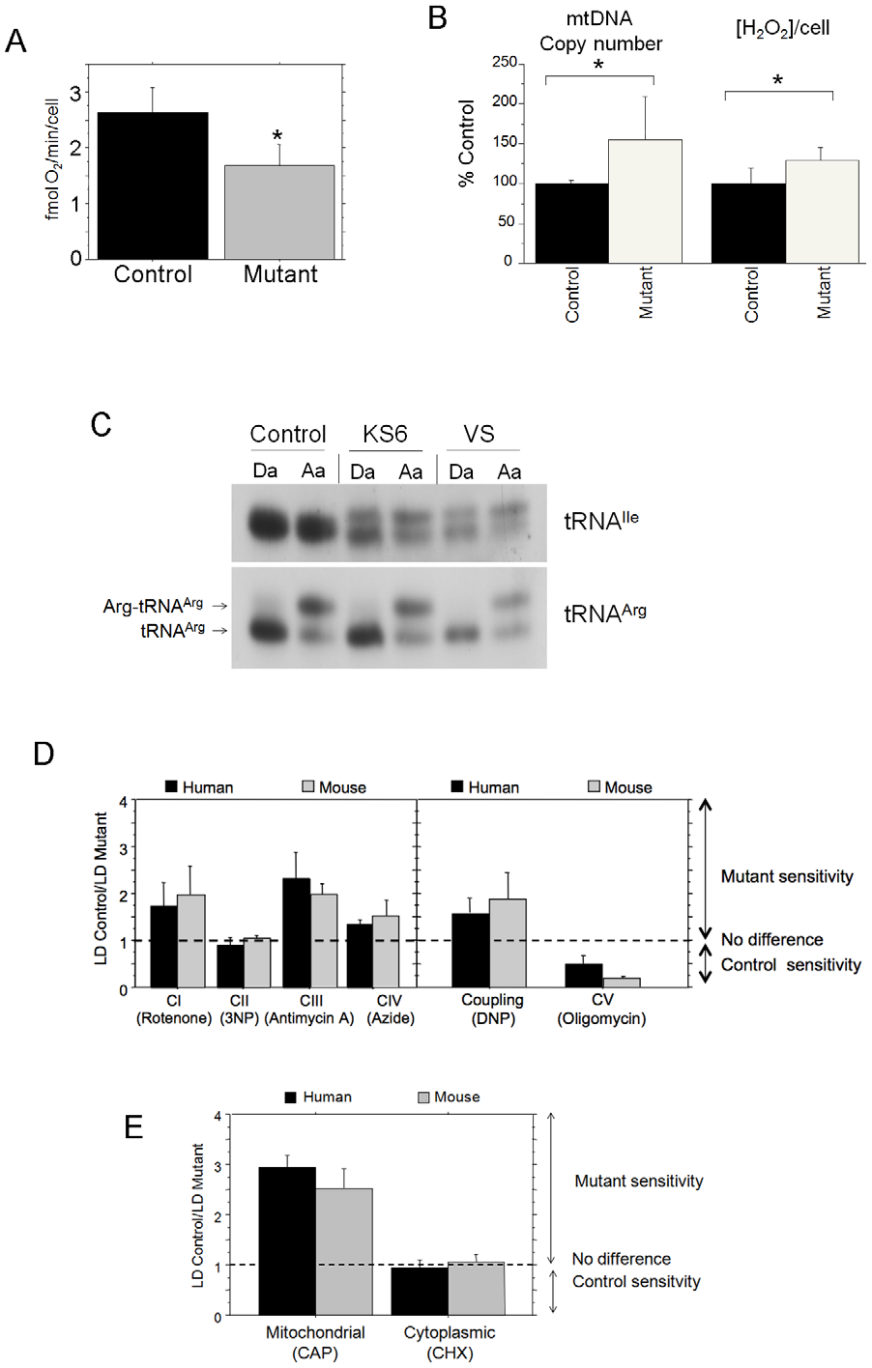
The human m.4290T>C mutation promotes the same molecular and phenotypic effects as the mouse mutation. A) Oxygen consumption rate in intact cells (n = 25 and 7, in controls and mutants, respectively p<0.0001). B) mtDNA copy number variation (n = 8 and 23 for control and mutants, respectively p = 0.0179) and H_2_O_2_ production between wild type and mutant cells n = 16 and 18 for control and mutants respectively, p<0.0001). C) Analysis of the aminoacylation capacity of mt-tRNA^Ile^ in human cells carrying the m.4290T>C mutation. The identification of the lower band as the uncharged tRNA was made by running in parallel a sample of deacylated tRNA and the quality of the samples was tested by hybridization with a different probe specific for mt-tRNA^Arg^. D) Relative ratio of LD_50_ for the indicated inhibitors of the different respiratory complexes and the uncoupling or the inhibition of mitochondrial ATP synthase, (Human cells: n≥3 in all cases but sodium azide (n = 2 for control cells). Mouse cells: see Figure 2F and 2G) E) Differential influence of CAP or cycloheximide in wild-type versus mutant cells viability (CAP n = 3 in both cases and Cycloheximide: n = 4 and 8 for control and mutant cells; p = 0.0109 in the case of CAP and p = 0.5536 for cycloheximide.). Data are given as the mean ± standard deviation of the mean. Asterisks indicate significant differences respect to each control, tested by ANOVA post-hoc Fisher PLSD (p<0.05). The control group is composed by transmitochondrial cybrids belonging to different mtDNA haplogroups whereas the mutant group is formed by two independent clones (VS and KS6) belonging to haplogroup U6 and harboring the m.4290T>C mutation in homoplasmic form.

In agreement with the results obtained in mouse cells, the human tRNA^Ile^ mutant caused reduced levels of respiration when compared to control (Figure 8A). More interesting, as shown in Figure 8B, both mtDNA copy number and H_2_O_2_ production were increased in human mutant cells as observed in mouse mutants. When human mitochondrial tRNAs isolated under acidic conditions were electrophoresed through acid gels and hybridized with mt-tRNA^Ile^ specific probes, a slower migrating band appeared in mutant samples (Figure 8C). This second band, which was not present in controls, remained even after strong deacylation treatment (pH = 8.5 and heating for 15 minutes at 95°C) and is identical to the slower moving band described above for the mouse mutant tRNA. In addition, as in mouse cells, we confirmed that the human tRNA^Ile^ mutation promotes a higher sensitivity of cell growth to complex I, III and IV inhibitors, as well as to uncouplers, without modification of the sensitivity to complex II inhibition (Figure 8D). Again, human mutant cells were more resistant than wild type to inhibition of ATPase by oligomycin (Figure 8D). Finally, human mutant cells were more sensitive to CAP but not to cycloheximide compared to wild type, as was observed in mouse cells (Figure 8E).

## Discussion

We describe here the generation and characterization of the first pathological mutation in a mitochondrial tRNA gene in mouse cells, a G to A transition at position 3739 within the mt-tRNA^Ile^ anticodon loop. We have carefully analyzed the phenotype induced by this mutation and established its deleterious and potentially pathogenic character. Our conclusion is supported by the following results:

- The m.3739G>A mutation in the *mt-Ti* is the only one found in the entire mtDNA of the mutant cell line.
- The mutation alters the secondary and/or the tertiary structure of the tRNA.
- The mutation impairs mitochondrial protein synthesis producing an alteration in the proportion of respiratory complexes and the accumulation of subcomplexes.
- Cells harboring the mutation show OXPHOS impairment and defective growth in galactose.

Thirteen mutations affecting *mt-Ti* gene have been reported to date in humans. Most of them seem to affect primarily tRNA biosynthesis, leading to a drop in its steady-state levels [41]. Four different mutations, which are placed in different regions of the cloverleaf structure of this tRNA, exert their effect on mutant tRNA biosynthesis by impairing the efficiency of its 3′-end maturation [8] and at least one, m.4269A>G, located within the tRNA acceptor stem, promotes tRNA instability both *in vivo* and *in vitro* [6] because of a reduction in binding affinity of this tRNA for elongation factor Tu [42]. Other mutations reduce the efficiency of aminoacylation [8]. Three of them, m.4290T>C, m.4291T>C and m.4295A>G, are located in the anticodon loop. The m.4295A>G causes hypertrophic cardiomyopathy [43], seems to affect 3′-end maturation [8], and promotes a 50% reduction in tRNA steady-state level [41]. The m.4291T>C transition has been associated with hypertension, hypercholesterolemia, and hypomagnesemia [44], but nothing is known about the molecular effects induced by this mutation. Finally, we report here that the human m.4290T>C, associated with progressive necrotizing encephalopathy [16], promotes an alternative folding of the tRNA with similar probability to the canonical folding. Two of these anticodon-loop mutations in human tRNA^Ile^ have been reported as homoplasmic (m.4290T>C and m.4291T>C) showing a variable penetrance that remains unexplained at the molecular level. In addition, a new mutation (m.4296G>A) corresponding to the one described here, has been found in humans associated with a degenerative encephalopathy (Rossmanith, W., personal communication).

The mouse mutation described here (m.3739G>A) is also located at the anticodon loop of the tRNA^Ile^, at the same position that the human m.4296G>A, and also induces a similar alternative folding of the tRNA to that promoted by the human m.4290T>C. This may be possible despite being in a different relative position of the tRNA because both may generate the same new base pairing in the anticodon-loop of the tRNA^Ile^ [16]. This alternative folded tRNA^Ile^ is not aminoacylated, representing a defective tRNA secondary/tertiary structure that cannot participate in protein synthesis and, therefore, reduces the amount of functional tRNA. Together with that, we have established that both mutations, in humans and in mouse cells, cause only a moderate defect in OXPHOS that is accompanied by accumulation of subcomplexes of the respiratory chain. Therefore, the mouse mutation seems to fully reproduce the molecular hallmarks of a described human mtDNA mutation affecting a tRNA. We would like to stress that both the human and the mouse mutations could reach homoplasmy because the OXPHOS deficiency they promote is moderate. Therefore, they represent an intriguing set of mitochondrial tRNA mutations with very variable penetrance than can cause no symptoms or, as it is the case of the tRNA^Ile^ mutation, a devastating neurological disease in members of the same family [16]. In particular, the mother was homoplasmic for the m.4290T>C mutation without showing any symptoms while her daughters suffered the diseases at different stages [16]. Understanding this phenomenon is critical in our attempt to develop therapeutic strategies for these diseases.

**Figure 9:**
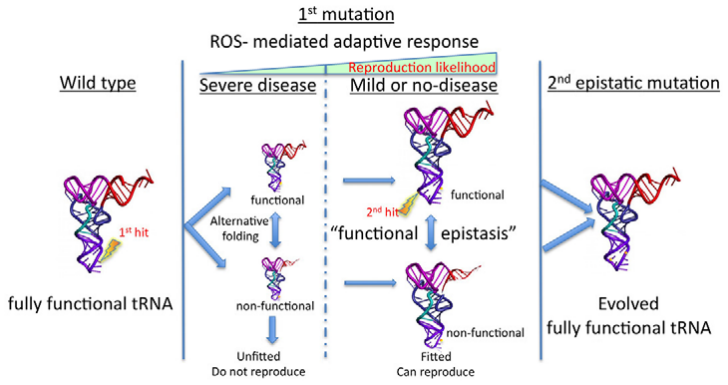
Modeling of the potential consequences of the “functional epistasis” on disease penetrance and sequence evolution of mitochondrial tRNAs. First, a mitochondrial tRNA (mt-tRNA) mutation occurs that disestablished the functional structure of the tRNA making a second but non-functional folding similarly feasible. If the mutation reaches homoplasmy substantially reduces the availability of functional tRNA and can compromise mitochondrial protein synthesis fidelity. As a consequence mitochondria biogenesis is triggered by a ROS-induced mechanism that is modulated by genetic and environmental factors. Depending of the amplitude of the compensatory mechanism, the disease would either be prevented (or substantially ameliorated) or declared. If prevented, the mutant mtDNA could be transmitted by the female germ-line to the descendants. Within the next generation and for each new individual the same options are open again, and therefore the mutation effectively reduces the fitness of their carriers by reducing the likelihood of reproduction and would be lost in a few generations. However, this scenario substantially increases the likelihood for the emergence of a second mutation in the same molecule, a true epistatic mutation, that can render the tRNA fully functional again and that would be definitively fixed.

Here we are proposing a model aimed to explain this behavior that we would like to call “functional epistasis model” (Figure 9). The concept of epistasis refers to the suppression of the effect of one gene by another or of one mutation by another. This is very relevant in evolutionary studies to understand the changes in gene sequences that may affect function. In particular, this has been studied in mammalian mitochondrial tRNAs [45]-[47]. One fundamental conclusion of these studies is that in numerous cases mammalian mitochondrial tRNAs has crossed low-fitness genotypes to reach isolated fitness peaks [47]. This has lead to the conclusion that simultaneous fixation of two alleles that are individually deleterious may be a common phenomenon at the molecular level in the evolution of mitochondrial tRNAs. But it still remains to be clarified if this compensatory evolution does proceed through rare intermediate variants that never reach fixation or not.

We believe that it may be possible to assimilate the homoplasmic tRNA mutations causing diseases together with their variable penetrance in humans with the rare intermediate variants required to cross the low fitness- valleys in tRNA evolution as follows (Figure 9):

First, a mitochondrial tRNA (mt-tRNA) mutation occurs that destabilizes the functional structure of the tRNA making a second but non-functional folding similarly feasible. If the mutation reaches homoplasmy, it substantially reduces the availability of functional tRNA and can compromise mitochondrial protein synthesis fidelity. As a consequence, an unbalance in the assembly of respiratory complexes induces a compensatory response by increasing mitochondrial biogenesis through a rise in the basal production of ROS (H_2_O_2_). As has been described for mouse mt-tRNA non-pathological variants [31], this response can be in some cases sufficient to compensate the deleterious effect of the mutation. In other cases, differences in the amplitude of this response modulated by gene context or environmental factors can render this compensatory response insufficient to prevent the expression of the disease phenotype (Figure 9). For example, the excess of ROS can also trigger the expression of ROS defenses and if this response is very efficient the activation of mitochondrial biogenesis would be blocked, the effect of the mutation would not be compensated and the disease would manifest. Conversely, if ROS defenses are less efficient, mitochondrial biogenesis would be activated and the amount of functionally folded tRNA can grow to a level that can substantially compensate the mitochondrial protein synthesis defect. In this case, the disease would be prevented or substantially ameliorated. The woman harboring this mutation would be able to reproduce and, in that way, transmit the homoplasmic mutation in the mtDNA to the descendants. Within the next generation and for each new individual the same options would be open and some carriers could develop a fatal early disease while others could be asymptomatic; therefore the mutation effectively reduces the likelihood of reproduction of the carrier woman and would be likely lost in a few generations. However, this scenario substantially increases the likelihood for the emergence of a second mutation in the same molecule, a true epistatic mutation that can render the tRNA fully functional again, and the new variant would behave as a neutral allele within the population. This would be a plausible mechanism to cross low-fitness valleys in the evolution of mt-tRNAs [47].

## Materials and Methods

### Cell lines and media

All the cell lines were grown in DMEM (GibcoBRL) supplemented with 5% FBS (fetal bovine serum, Gibco BRL). MtDNA-less mouse cells (ρ^0^L929^neo^ and ρ^0^L929^puro^) were generated by long-term growth of L929 mouse cell line in the presence of high concentrations of Ethidium Bromide and transfection with the neocassette-containing plasmid pcDNA3.1 (Invitrogen) as previously described [34] or the purocassette-containing plasmid pBABE puro. TmBalb/cJ cells were generated by transference of mitochondria from platelets to ρ^0^L929^neo^ cells as described elsewhere [34], [48]. mB77 cells were derived by random mutagenesis of TmBalb/cJ cells using TMP (4,5′,8-trimethylpsoralen) and UV light as previously described [33] and harbor an A to G transition at position 3739 at *mt-Ti* gene. Balb/cJp1 and mB77p18 cells were generated by TmBalb/cJ or mB77 cytoplasts transference to ρ^0^L929^puro^ cells. Transmitochondrial cell lines were isolated by growing the cell population in DMEM supplemented with 5% dFBS and 10 µg/ml of puromycin (SIGMA). When indicated, cells were cultured in the presence of 5 mM NAC for a week or 1mM Tiron for 72 hours.

### DNA analysis

Total DNA from cell lines was extracted using standard procedures. The complete mtDNA was amplified in 24 overlapping 800-1,000 bp-long PCR fragments using a multifunctional robot (Genesis 150 TECAN, Crailsheim) as previously described [49]. Primers were designed using the reference sequence (NC_005089) [29].

### RFLP analysis

To confirm the presence of the mutation, RFLP analysis was achieved. See Table S1 for primer sequences. The primer-generated mutation together with the A3739 mutant version creates two recognition sites for Tru9I and produces three bands of 63, 51 and 41 bp upon digestion with this enzyme. The restriction site that produces the 63 and 41 bp bands is disrupted when the WT version G3739 is present and a new band of 104 bp appears. Therefore, an internal control for full digestion with Tru9I is present in the analysis. Fragments were analyzed by electophoresis in a 10% polyacrylamide gel.

### Growth measurements

Growth capacity was determined by plating 5*10^4^ cells on 12 wells test plates in 2 ml of the appropriate medium (DMEM, which contains 4.5 mg of glucose/ml supplemented with 5% FBS, or DMEM lacking glucose and containing 0.9 mg of galactose/ml, supplemented with 5% dFBS), incubating them at 37°C for 5 days and performing cell counts at daily intervals.

### Oxygen consumption measurements

O_2_ consumption determinations in intact or in digitonine-permeabilized cells were carried out in an oxytherm Clark-type electrode (Hansatech) as previously described [50] with small modifications [34].

### Enzymatic activity measurements

Mitochondria were isolated as described previously [51] and the different enzymatic activities were assessed by spectrophotometry. Citrate synthase and complexes I, II, III and IV activities were measured in isolated mitochondria as described before [32], [52].

### Cell viability assays

Effect of different inhibitors on the viability of control and mutant cells was evaluated using the MTT reduction assay according to Mosmann et al [53]. This is an indirect way of measuring cell viability in which mitochondrial dehydrogenases of viable cells reduce the yellowish water-soluble MTT to water-insoluble formazan crystals. These crystals are solubilized with dimethyl sulfoxide (DMSO) and optical density (OD) is read on an ELISA reader (TECAN) at 570 nm.

Briefly, cells were plated into 96-well microtiter plates at a density of 2.5*10^3^ cells per ml, in the case of human cells, and 5*10^3^ cells per ml when analyzing mouse cells. Then, cells were cultured for 3 days to be allowed to reach exponential growth rate before drug addition. Afterward, cells were cultured in galactose containing medium with inhibitors for 48 hours (for inhibitory concentration ranges see Table S2). After drug exposition, cells were fed with fresh galactose-containing medium and allowed to grow for 2 population doubling times. At the end of the recovery period, plates were incubated with fresh medium and MTT for 4 hours in a humidified atmosphere at 37°C, formazan crystals solubilized and OD at 570 nm read. The results obtained were given as relative values to the untreated control in percent and lethal dose 50 was determined as the drug concentration required to reduce the absorbance to half that of the control. All experiments were performed at least in triplicate.

### Mitochondrial DNA copy number quantification

MtDNA quantification was performed by real-time PCR using an ABI PRISM 7000 Sequence Detector System (AB Applied Biosystems) and Platinum SYBR Green qPCR SuperMix-UDG (Invitrogen). Total cellular DNA was used as template and was amplified with specific oligodeoxynucleotides for *mtCo2* (from position 7037 to 7253 in mouse (NC_005089) and from 7859 to 7927 in human samples (NC_012920)) and *SdhA* (from position 1026 to 1219 in mouse (AK049441) and from position 224 to 295 (AF171018) in human DNA). mtDNA copy number per cell was calculated using *SdhA* amplification as a reference for nuclear DNA content as previously reported [31]. See Table S1 for primer sequences.

### Determination of hydrogen peroxide production

Production of hydrogen peroxide was measured in cultured cells grown in the absence or in the presence of NAC for 1 week as previously described [31]. Briefly, 100,000 cells were incubated at 37°C for 30 minutes in the presence of 100 µM 2′,7-Dichlorodihydrofluorescein diacetate (2,7-DCFH_2_-DA, Fluka). Then, cells were collected and the reaction was stopped in an ice-bath for 5 minutes. After that, cells were disrupted by treatment with Triton X-100 (2%) and centrifuged at 2,500 g for 20 minutes at 4°C. The supernatant was used to measure fluorescence emission (excitation at 485 nm and emission at 535 nm) in a TECAN Spectrafluor plus. The amount of hydrogen peroxide produced was then calculated using a standard curve of 2,7-DCF in which 1 µM of 2,7-DCF represented 1 µM of hydrogen peroxide.

### Mitochondrial protein synthesis analysis

Labeling of mtDNA-encoded proteins was performed with [^35^S]-methionine in intact cells as described elsewhere for 1 hour [54]. In the pulse-chase experiments, labeling was carried out in the presence of cycloheximide to inhibit cytoplasmic protein synthesis for 2 hours. Then the drug and the label were removed and the incorporation of the labeled proteins in fully assembled complexes was followed after 48 hours of chase.

### Assembled protein detection by western blot

Estimation of the relative level of the assembled respiratory complexes in cell lines was performed by Blue-Native electrophoresis (BNGE) followed by western blot as described before [32].

Confirmation of the presence of subcomplexes in mutant cells was carried out using two-dimensional BNGE/SDS-PAGE. These filters were sequentially probed with specific antibodies: anti-NDUFB6 (complex I) anti-SDHB (ISP30) or anti-SDHA (Fp70) (complex II), anti-Core 2 (complex III), and anti-CO I (complex IV) from Molecular Probes.

### Mitochondrial RNA isolation

The mitochondrial fraction, isolated from cell cultures as described previously [51], was suspended in 10 mM Tris-HCl (pH = 7.4), 0.15 M NaCl, 1 mM EDTA, and incubated for 15 minutes at 37°C in the presence of proteinase k (200 µg/ml), SDS (2%) and RNAse-free DNAse (Roche). Then, total mitochondrial RNAs were extracted with an equal volume of phenol-chloroform-isoamyl alcohol (25∶25∶1), and then precipitated with ethanol [38], [39]. In the experiments in which aminoacyl-tRNA complexes had to be preserved, isolation of mitochondrial fraction was followed by extraction of RNAs under acid conditions as previously described [38], [39].

### Quantification of the mitochondrial mt-tRNA^Ile^

The relative content of mt-tRNA^Ile^ was determined as described elsewhere [4]. Briefly, total mitochondrial RNA preparations were electrophoresed through a 10% polyacrylamide-7 M urea gel in Tris-borate-EDTA buffer (after heating the sample at 70°C for 10 minutes) and then electroblotted onto a Zeta-probe membrane (Bio-Rad) for hybridization analysis with specific oligodeoxynucleotides probes. These probes were 5′-end labelled through T4 polynucleotide kinase (Promega) reaction using [γ^32^P]-dATP (Amersham). For the detection of mt-tRNA^Ile^ and mt-tRNA^Gly^, oligodeoxynucleotides specific for each tRNA were used (Table S1).

The hybridization reactions were carried out in a mixture of 6x SSC, 0.1% sodium pyrophosphate, 5x Denhardt's solution, 0.1% SDS and 250 µg of salmon sperm DNA per ml, for 4 h at 37°C. After hybridization, the membranes were washed twice for 10 min in 2x SSC-0.1% SDS at 37°C.

### Sequencing of 5′- and 3′- ends mt-tRNA^Ile^

The 5′ and 3′ ends of the mt-tRNA^Ile^ from TmBalb/cJ and from the mutant cell line mB77 were sequenced after cDNA synthesis, PCR amplification and cloning as described elsewhere [11]. Briefly, mt-tRNA fractions were obtained by polyacrylamide gel electrophoresis as previously detailed [39] and circularized by incubation in the presence of T4-RNA ligase (Promega). Then, a complementary DNA chain of mt-tRNA^Ile^ was synthesized by reverse transcriptase using 1^st^ Strand cDNA Synthesis Kit for RT-PCR (AMV) from Roche with the specific oligodeoxynucleotide tIle1: TATCAAAGTAATTCTTTTATC. The mt-tRNA^Ile^ fragment was then synthesized by PCR using the primers tIle1 and tIle2 (AGTAAATTATAGAGGTTCAAG) and cloned in the TA Cloning vector (Invitrogen).

### Radiolabeled primer extension of mt-tRNAIle 5′- and 3′- ends

To confirm the proper 3′-end processing and CCA addition in the mutant tRNA^Ile^, radiolabeled primer extension was performed. Thus, the mt-tRNA^Ile^ fragment synthesized as described above was used as template and the 5′ end ^32^P-labeled tIle-PE oligodeoxynucleotide was used as a primer: tIle-PE: TTCAAGCCCTCTTATTTCTA. Nucleotide concentrations were: 50 µM dCTP and 500 µM ddATP. For control purposes we used a synthetic mt-tRNA^Ile^ (Dharmacon). The radioactive signal was developed using the Personal Molecular Imager system from BIO-RAD and analyzed with 1-D Analysis software Quantity One (BIO-RAD).

### Identification of mt-tRNAs charged and uncharged forms

The mtRNA fraction isolated under acid conditions was electrophoresed at 4°C through a 10% polyacrylamide-0 to 8 M urea gel in 0.1 M sodium acetate (pH = 5) at 100–200 V and then electroblotted and the mt-tRNAs charged and uncharged forms were identified by sequential hybridization with specific probes as described above. (Primer sequences in Table S1).

### *In organello* aminoacylation assays

For *in organello* aminoacylation, mitochondria were purified as described before [51] and the mitochondrial pellets were incubated in the appropriated medium as previously detailed [39]. Briefly, the isolated organelles (∼1 mg of protein) were incubated in 0.5 ml buffer containing 10 mM Tris-HCl, pH = 7.4, 100 mM KCl, 5 mM MgCl_2_, 10 mM K_2_HPO_4_, 50 µM EDTA, 1 mM ADP, 10 mM glutamate, 2.5 mM malate, 25 mM sucrose, 75 mM sorbitol, 1 mg/ml BSA, a mixture of all aminoacids (except the labeled one) to a concentration of 10 µM each and 75 µCi of a ^3^H-labeled aminoacid (Amersham). Incubation was carried out at 37°C for 15 minutes in a rotary shaker (12 rpm) and analysis of aminoacylation was performed as described above.

### Statistical analysis

The differences between control and mutant cell lines for the various parameters analyzed were assessed by analysis of variance (ANOVA). Paired haplotype differences were assessed by the post hoc Fisher's protected least significant difference test (PLSD). All tests and calculations were done with the statistical package StatView 5.0 for Macintosh (SAS Institute, Inc.).

## Supporting Information

Figure S1 Analysis of the biogenetic response induced by the mutation. A) mtDNA copy number variation between wild type and mutant cells (n = 23, 21, 13 and 11 for TmBalb/cJ, mB77, Balp1 and mB77p18 respectively and p<0.0001 between each mutant and its control) B) H_2_O_2_ production by wild type and mutant cells (n = 17, 13, 8 and 6 for TmBalb/cJ, mB77, Balbp1 and mB77p18 respectively and p<0.005 between each mutant cell line and its control). C) Influence of N-acetyl cysteine (NAC) on the H_2_O_2_ production and mtDNA copy number in wild type and mutant cells (n = 3 in all cases for H_2_O_2_ production (left) and n = 3 in all cell lines except in mB77 where n = 4 in mB77 for mtDNA copy number (right)). D) Influence of NAC on the respiration activity of permeabilized cells with different substrates (n =  5, 4, 3 and 5 fo5 TmBalb/cJ, mB77, Balbp1 and mB77p18 respectively). All values are given as mean ± SD of the mean. Asterisks indicate significant differences respect to each control, tested by ANOVA post-hoc Fisher PLSD (p<0.05).
(0.15 MB DOC)

Figure S2 m.3739G>A mutation and mt-tRNA^Ile^ precursor processing. It has been reported that some pathogenic mutations in mt-tRNA^Ile^ affect steps in tRNA maturation including 3′-end processing and CCA addition [1], [2]. To analyze the possible effect of m.3739G>A mutation on mt-tRNA^Ile^ precursor processing, several cDNA clones derived from circularized mt-tRNA^Ile^ from wild type and mutant cell lines were sequenced [3]. Thus, 14 out of 17 sequences from control cells and 11 out of 18 from mutant cells showed the expected 3′CCA and 5′ ends (See alignments and table below). Some of the remaining sequences are likely due to artifacts where the oligodeoxynucleotide used for cDNA synthesis was ligated to the 5′-end of the tRNA. The gene encoding the mt-tRNA^Ile^ overlaps two nucleotides with the 3′ end of the *mt-Nd1* gene and three nucleotides with the 5′ end of the gene encoding for the tRNA^Gln^. We believe that RNAs derived from the processing of tRNA^Gln^ and ND1 mRNA explain the finding of this proportion of circularized products with the lack of 3′ and 5′ portions of the tRNA^Ile^. In summary, since the major proportion of molecules showed a proper maturation of the 3′ and 5′ and CCA addition, we conclude that no major defect in the processing of the mt-tRNA^Ile^ can be attributed to the mutation.
(0.20 MB DOC)

Table S1 Primer sequences.
(0.05 MB DOC)

Table S2 Inhibitor concentration ranges.
(0.05 MB DOC)
