# Supplementary material for: Evolution Meets Disease: Penetrance and Functional Epistasis of Mitochondrial tRNA Mutations
Source: PLoS Genet. 2011 Apr 21;7(4):e1001379. doi: 10.1371/journal.pgen.1001379 (PMC3080857; doi:10.1371/journal.pgen.1001379)
Supplement: Figure S1 — Analysis of the biogenetic response induced by the mutation. A) mtDNA copy number variation between wild type and mutant cells (n = 23, 21, 13 and 11 for TmBalb/cJ, mB77, Balp1 and mB77p18 respectively and p<0.0001 between each mutant and its control) B) H2O2 production by wild type and mutant cells (n = 17, 13, 8 and 6 for TmBalb/cJ, mB77, Balbp1 and mB77p18 respectively and p<0.005 between each mutant cell line and its control). C) Influence of N-acetyl cysteine (NAC) on the H2O2 production and mtDNA copy number in wild type and mutant cells (n = 3 in all cases for H2O2 production (left) and n = 3 in all cell lines except in mB77 where n = 4 in mB77 for mtDNA copy number (right)). D) Influence of NAC on the respiration activity of permeabilized cells with different substrates (n = 5, 4, 3 and 5 fo5 TmBalb/cJ, mB77, Balbp1 and mB77p18 respectively). All values are given as mean ± SD of the mean. Asterisks indicate significant differences respect to each control, tested by ANOVA post-hoc Fisher PLSD (p<0.05). (0.15 MB DOC) [file pgen.1001379.s001.doc]

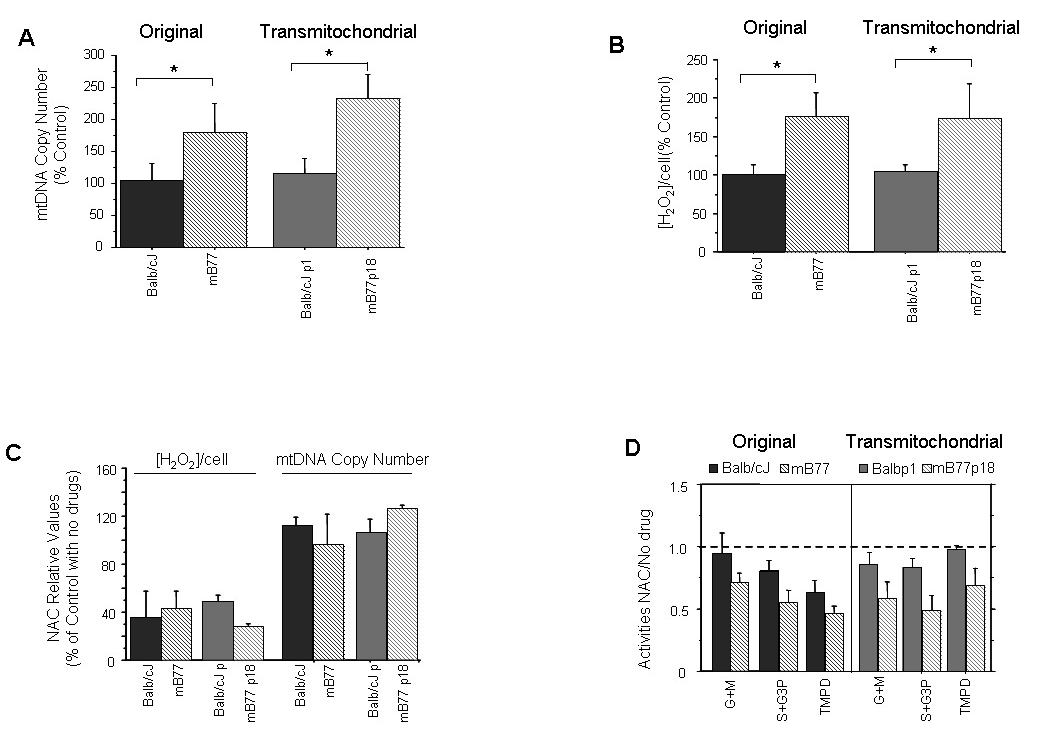
**Supplementary Results**

**Figure S1.-** Analysis of the biogenetic response induced by the mutation. A) mtDNA copy number variation between wild type and mutant cells (n=23, 21, 13 and 11 for TmBalb/cJ, mB77, Balp1 and mB77p18 respectively and p<0.0001 between each mutant and its control) B) H_2_O_2_ production by wild type and mutant cells (n=17, 13, 8 and 6 for TmBalb/cJ, mB77, Balbp1 and mB77p18 respectively and p<0.005 between each mutant cell line and its control). C) Influence of N-acetyl cysteine (NAC) on the H_2_O_2_ production and mtDNA copy number in wild type and mutant cells (n=3 in all cases for H_2_O_2_ production (left) and n=3 in all cell lines except in mB77 where n=4 in mB77 for mtDNA copy number (right)). D) Influence of NAC on the respiration activity of permeabilized cells with different substrates (n= 5, 4, 3 and 5 fo5 TmBalb/cJ, mB77, Balbp1 and mB77p18 respectively). All values are given as mean ± SD of the mean. Asterisks indicate significant differences respect to each control, tested by ANOVA post-hoc Fisher PLSD (p<0.05).
