# Supplementary material for: Evolution Meets Disease: Penetrance and Functional Epistasis of Mitochondrial tRNA Mutations
Source: PLoS Genet. 2011 Apr 21;7(4):e1001379. doi: 10.1371/journal.pgen.1001379 (PMC3080857; doi:10.1371/journal.pgen.1001379)
Supplement: Figure S2 — m.3739G>A mutation and mt-tRNAIle precursor processing. It has been reported that some pathogenic mutations in mt-tRNAIle affect steps in tRNA maturation including 3′-end processing and CCA addition [1], [2]. To analyze the possible effect of m.3739G>A mutation on mt-tRNAIle precursor processing, several cDNA clones derived from circularized mt-tRNAIle from wild type and mutant cell lines were sequenced [3]. Thus, 14 out of 17 sequences from control cells and 11 out of 18 from mutant cells showed the expected 3′CCA and 5′ ends (See alignments and table below). Some of the remaining sequences are likely due to artifacts where the oligodeoxynucleotide used for cDNA synthesis was ligated to the 5′-end of the tRNA. The gene encoding the mt-tRNAIle overlaps two nucleotides with the 3′ end of the mt-Nd1 gene and three nucleotides with the 5′ end of the gene encoding for the tRNAGln. We believe that RNAs derived from the processing of tRNAGln and ND1 mRNA explain the finding of this proportion of circularized products with the lack of 3′ and 5′ portions of the tRNAIle. In summary, since the major proportion of molecules showed a proper maturation of the 3′ and 5′ and CCA addition, we conclude that no major defect in the processing of the mt-tRNAIle can be attributed to the mutation. (0.20 MB DOC) [file pgen.1001379.s002.doc]

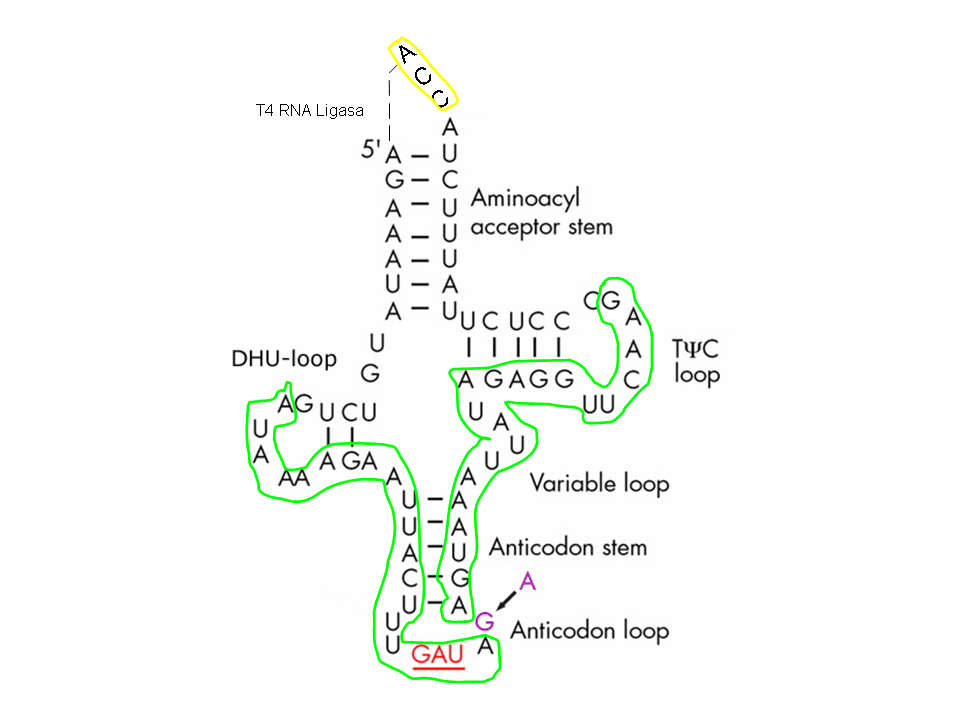


**Legend**

TT pCR2.1 Cloning ends

NNNNNNNNN PCR primers.

CCA Expected 3’CCA and 5’ ends

**Figure S2.- m.3739G>A mutation and mt-tRNA^Ile^ precursor processing**

It has been reported that some pathogenic mutations in mt-tRNA^Ile^ affect steps in tRNA maturation including 3’-end processing and CCA addition . To analyze the possible effect of m.3739G>A mutation on mt-tRNA^Ile^ precursor processing, several cDNA clones derived from circularized mt-tRNA^Ile^ from wild type and mutant cell lines were sequenced . Thus, 14 out of 17 sequences from control cells and 11 out of 18 from mutant cells showed the expected 3’CCA and 5’ ends (See alignments and table below). Some of the remaining sequences are likely due to artifacts where the oligodeoxynucleotide used for cDNA synthesis was ligated to the 5’-end of the tRNA. The gene encoding the mt-tRNA^Ile^ overlaps two nucleotides with the 3’ end of the *mt-Nd1* gene and three nucleotides with the 5’ end of the gene encoding for the tRNA^Gln^. We believe that RNAs derived from the processing of tRNA^Gln^ and ND1 mRNA explain the finding of this proportion of circularized products with the lack of 3’ and 5’ portions of the tRNA^Ile^. In summary, since the major proportion of molecules showed a proper maturation of the 3’ and 5’ and CCA addition, we conclude that no major defect in the processing of the mt-tRNA^Ile^ can be attributed to the mutation.

Summary Table

| mtDNA | **Number of sequenced clones** | **Number of modified sequences** | % Modified sequences |
| --- | --- | --- | --- |
| Control  Mutant | 17  18 | 3  7 | 17,6  38,8 |

**Control cells: Right Sequences**

34_B9_M13FW TTAGTAAATTATAGAGGTTCAAGCCCTCTTATTTCTACCAAGAAATATGTCTGATAAAAGAATTACTTTGATAA

36_B10_M13FW TTAGTAAATTATAGAGGTTCAAGCCCTCTTATTTCTACCAAGAAATATGTCTGATAAAAGAATTACTTTGATAA

30_B7_M13FW TTAGTAAATTATAGAGGTTCAAGCCCTCTTATTTCTACCAAGAAATATGTCTGATAAAAGAATTACTTTGATAA

49_B5_M13FW TTAGTAAATTATAGAGGTTCAAGCCCNCCTCNTTCTACCAAGAAATATGTCTGATAAAAGAATTACTTTGATAA

05_B2_M13RV CTAGTAAATTATNGAGGTTCAAGCCCTCTTATTTCTACCAAGAAATATCTCTGATAAAAGAATTACTTTGATAA

48_B4_M13FW TTAGTAAATTATAGAGGTTCAAGCCCTCTTATTTCTACCAAGAAATATCTCTGATAAAAGAATTACTTTGATAA

22_BP1_M13FW TTAGTAAATTATAGAGGTTCAAGCCCTCTTATTTCTACCAAGAAATATGTCTGATAAAAGAATTACTTTGATAA

23_BP3_M13FW TTAGTAAATTATAGAGGTTCAAGCCCTCTTATTTCTACCAAGAAATATGTCTGATAAAAGAATTACTTTGATAA

38_BP6_M13FW TTAGTAAATTATAGAGGTTCAAGCCCTCTTATTTCTACCAAGAAATATGTCTGATAAAAGAATTACTTTGATAA

44_BP9_M13FW TTAGTAAATTATAGAGGTTCAAGCCCTCTTATTTCTACCAAGAAATATGTCTGATAAAAGAATTACTTTGATAA

46_BP10_M13FW TTAGTAAATTATAGAGGTTCAAGCCCTCTTATTTCTACCAAGAAATATGTCTGATAAAAGAATTACTTTGATAA

24_BP4_M13FW TTAGTAAATTATAGAGGTTCAAGCCCTCTTATTTCTACCAAGAAATATGTCTGATAAAAGAATTACTTTGATAA

41_BP8_M13RW TTAGTAAATTATAGAGGTTCAAGCCCTCTTATTTCTACCAAGAAATATCTCTGATAAAAGAATTACTTTGATAA

25_BP5_M13F TTAGTAAATTATAGAGGTTCAAGCCCTCTTATTTCTACCAAGAAATATGTCTGATAAAAGAATTACTTTGATAA

*********** ************* * * **************** *************************

**Control cells: Modified sequences**

31_B8_M13RV TTAGTAAATTATAGAGGTTCAAGCCCTCTTATTACCA-C--------------GATAAAAGAATTACTTTGATAAA

40_BP7_M13FW TTAGTAAATTATAGAGGTTCAAGCCCTCTTATTT----C--------------GATAAAAGAATTACTTTGATAAA

04_B1_M13RV TTAGTAAATTACNTAGGTTCAAGCCCTCTTATTT--ACCCAA-----------GATAAAAGAATTACTTTGATAGA

*********** ******************* * ********************* *

**Mutant cells: Right sequences**

50_771_M13FW TTAG--TAAATTATAGAGGTTCAAGCCCTCTTATTTTTACCAAGAAATATGTCTGATAAAAGAATTACTTTGATAAA

14_7713_M13FW TTAG--TAAATTATAGAGGTTCAAGCCCTCTTATTTTTACCAAGAAATATGTCTGATAAAAGAATTACTTTGATAAA

51_772_M13FW TTAG--TAAATTATAGAGGTTCAAGCCCTCTTATTTTTACCAAGAAATATGTCTGATAAAAGAATTACTTTGATAAA

26_7719_M13FW TTAG--TAAATTATAGAGGTTCAAGCCCTCTTATTTTTACCAAGAAATATGTCTGATAAAAGAATTACTTTGATAAA

28_7720_M13FW TTAG--TAAATTATAGAGGTTCAAGCCCTCTTATTTTTACCAAGAAATATGTCTGATAAAAGAATTACTTTGATAAA

15_7714_M13RV TTAG--TAAATTATAGAGGTTCAAGCCCTCTTATTTTTACCAAGAAATATGTCTGATAAAAGAATTACTTTGATAAA

19_7716_M13RV TTAG--TAAATTATAGAGGTTCAAGCCCTCTTATTTTTACCAAGAAATATGTCTGATAAAAGAATTACTTTGATAAA

13_77P1_M13RV TTAG--TAAATTATAGAGGTTCAAGCCCTCTTATTTTTACCAAGAAATATGTCTGATAAAAGAATTACTTTGATAAA

54_775_M13FW TCGGCTTAAATTATAGAGGTTCAAGCCCTCTTATTTTTACCAAGAAATATGTCTGATAAAAGAATTACTTTGATAAA

27_77-9_M13FW TTAG--TAAATTATAGAGGTTCAAGCCCTCTTATTTTTACCAAGAAATATGTCTGATAAAAGAATTACTTTGATAAA

07_77-8_M13RV TTAG--TAAATTANAGAGGTTCAAGCCCTCTTATTTTTACCAAGAAATATGTCTGATAAAAGAATTACTTTGATAAA

* ******* ********************************** * **************************

**Mutant Cells: Modified Sequences**

11_77-12_M13RV TTAGTAAATTATAGAGGTTCAAGCCCTCTTATTTCTA-----CCAT--------GATAAAAGAATTACTTTGATAAA

12_77p-8_M13RV TTAGTAAATTATNGAGGTTCAAGCCCTCTTA-----A-----AAAA--------GATAAAAGAATTACTTTGATAAA

17_7715_M13RV TTAGTAAATTATAGAGGTTCAAGCCCTCTTATTAAAA-----AAATAT--CTCTGATAAAAGAATTACTTTGATAAA

24_7718_M13FW TTAGTAAATTATAGAGGTTCAAGCCCTCTTATTTCTACCGAGAAATAT--GTCTGATAAAAGAATTACTTTGATAAA

53_774_M13FW TTAGTAAATTATAGAGGTTCAAGCCCTCTTATT---------GAAT--------GATAAAAGAATTACTTTGATAAA

52_773_M13FW TTAGTAAATTATAGAGGTTCAAGCCCTCTTATTTCTA--------------CCAGATAAAAGAATTACTTTGATAAA

22_7717_M13FW TTCACACAGGAAACAGCTATGACCATGATTACGCCAAGCTTGGTACCGAGCTCGGATCCACTAGTAACGGCCGCCAG

** * * * ** * * * *** *** * * * ** *

### SUPPLEMENTARY REFERENCES:

1. Levinger L, Giege R, Florentz C (2003) Pathology-related substitutions in human mitochondrial tRNA(Ile) reduce precursor 3' end processing efficiency in vitro. Nucleic Acids Res 31: 1904-1912.

2. Tomari Y, Hino N, Nagaike T, Suzuki T, Ueda T (2003) Decreased CCA-addition in human mitochondrial tRNAs bearing a pathogenic A4317G or A10044G mutation. J Biol Chem 278: 16828-16833.

3. Guan MX, Enriquez JA, Fischel-Ghodsian N, Puranam RS, Lin CP, et al. (1998) The deafness-associated mitochondrial DNA mutation at position 7445, which affects tRNASer(UCN) precursor processing, has long-range effects on NADH dehydrogenase subunit ND6 gene expression. Mol Cell Biol 18: 5868-5879.
