# Supplementary material for: Evolution Meets Disease: Penetrance and Functional Epistasis of Mitochondrial tRNA Mutations
Source: PLoS Genet. 2011 Apr 21;7(4):e1001379. doi: 10.1371/journal.pgen.1001379 (PMC3080857; doi:10.1371/journal.pgen.1001379)
Supplement: Table S1 — Primer sequences. (0.05 MB DOC) [file pgen.1001379.s003.doc]

**Supplementary Table 1.- Primer Sequences**

*A) Primers used for m.3739G>A RFLP analysis*

| ***Oligo Name*** | ***Gene*** | ***Position*** | ***Sequence (5´ to 3´)*** |
| --- | --- | --- | --- |
| **3696-Fw*** | *mt-Ti* | 3696–3737 | CCATACATATAGAAATATGTCTGATAAAAGAATTACTTTGtT |
| **3817-Rev** | *mt-Ti* | 3817-3850 | CTTACTATTAGGATAAGGTGTTTAGGTAGCACGG |

(*)This primer contains a missmatch, indicated by the lower case letter, to generate internal restriction sites

*B) Primers used for mtDNA copy number quantification*

| ***Oligo Name*** | ***Gene*** | ***Species*** | ***Position*** | ***Sequence (5´ to 3´)*** |
| --- | --- | --- | --- | --- |
| **Co2-Fw** | *mt-Co2*  NC_005089 | Mouse | 7037-7052 | CTACAAGACGCCACAT |
| **Co2-Rev** |  |  | 7253-7238 | GAGAGGGGAGAGCAAT |
| **SdhA-Fw** | *SdhA*  AK049441 |  | 1026-1043 | TACTACAGCCCCAAGTCT |
| **SdhA-Rev** |  |  | 1219-1202 | TGGACCCATCTTCTATGC |
| **hCo2-Fw** | *MT-CO2*  NC_012920 | Human | 7858-7877 | CGATCCCTCCCTTACCATCA |
| **hCo2-Rev** |  |  | 7904-7926 | CCGTAGTCGGTGTACTCGTAGGT |
| **hSdhA-Fw** | *SDHA*  AF171018 |  | 224-244 | TCTCCAGTGGCCAACAGTGTT |
| **hSdhA-Rev** |  |  | 276-295 | GCCCTCTTGTTCCCATCAAC |

*C) Primers used for mt-tRNAs detection and quantification*

| ***Oligo Name*** | ***Gene*** | ***Species*** | ***Position*** | ***Sequence (5´ to 3´)*** |
| --- | --- | --- | --- | --- |
| **Leu^UUR^** | *mt-Tl1* | Mouse | 2730-2750 | TATTAGGGAGAGGATTTGAAC |
| **Ile** | *mt-Ti* |  | 3707-3731 | GTAATTCTTTTATCAGACATATTTC |
| **Trp** | *mt-Tw* |  | 4993-5016 | CAGAAGTTAAACTTGTGTGTTTTC |
| **Gly** | *mt-Tg* |  | 9422-9446 | GGTTTATTCAGAATCTACTAATTGG |
| **Arg** | *mt-Tr* |  | 9849-9873 | GGTAATTATGAACATCATCATAATC |
| **h-Ile** | *MT-TI* | Human | 4311-4331 | TAGAAATAAGGGGGTTTAAGC |
| **h-Arg** | *MT-TR* |  | 10452-10469 | TGGTTGGTAAATATGATTATC |

*D) Primers used for mt-tRNA^Ile^ 5’ and 3’ ends analysis*

| ***Oligo Name*** | ***Gene*** | ***Position*** | ***Use*** | ***Sequence (5´ to 3´)*** |
| --- | --- | --- | --- | --- |
| **tIle1** | *mt-Ti* | 3718-3738 | Amplification | TATCAAAGTAATTCTTTTATC |
| **tIle2** | *mt-Ti* | 3740-3760 | Amplification | AGTAAATTATAGAGGTTCAAG |
| **tIle-PE** | *mt-Ti* | 3755-3774 | Primer Extension | TTCAAGCCCTCTTATTTCTA |
