# Supplementary material for: Evolution Meets Disease: Penetrance and Functional Epistasis of Mitochondrial tRNA Mutations
Source: PLoS Genet. 2011 Apr 21;7(4):e1001379. doi: 10.1371/journal.pgen.1001379 (PMC3080857; doi:10.1371/journal.pgen.1001379)
Supplement: Table S2 — Inhibitor concentration ranges. (0.05 MB DOC) [file pgen.1001379.s004.doc]

**Table S2.- Inhibitor concentration ranges**

| Target | Inhibitor | Species | Concentration Range |
| --- | --- | --- | --- |
| **Mitochondrial Protein Synthesis** | Chloramphenicol | Mouse | 0-640 µg/ml |
|  |  | Human | 0-160 µg/ml |
|  | Pentamidine | Mouse | 0-60 µg/ml |
|  |  | Human | 0-3 µg/ml |
| **Cytoplasmic Protein Synthesis** | Cycloheximide | Mouse | 0-3 µg/ml |
|  |  | Human | 0-3 µg/ml |
| **Complex I** | Rotenone | Mouse | 0-200 nM |
|  |  | Human | 0-50 nM |
| **Complex II** | 3-Nitropropionic Acid | Mouse | 0-40 mM |
|  |  | Human | 0-10 mM |
| **Complex III** | Antimycin A | Mouse | 0-800 nM |
|  |  | Human | 0-400 nM |
| **Complex IV** | Sodium Azide | Mouse | 0-2 mM |
|  |  | Human | 0-0.5 mM |
| **ATP Synthase** | Oligomycin | Mouse | 0-16 nM |
|  |  | Human | 0-4 nM |
| **Coupling** | Dinitrophenol | Mouse | 0-650 µM |
|  |  | Human | 0-450 µM |
